# Lymphadenectomy in ovarian cancers: a meta-analysis of hazard ratios from randomized clinical trials

**DOI:** 10.1186/s12957-022-02835-4

**Published:** 2022-11-22

**Authors:** Roli Purwar, Rakesh Ranjan, Kishan Soni, Manoj Pandey, Satyanshu K. Upadhyay, Esha Pai, Tarun Kumar

**Affiliations:** 1 Department of Surgical Oncology, Institute of Medical Sciences, Banaras Hindu University, Varanasi, 221005 India; 2 Department of Science and Technology (DST), Centre for Interdisciplinary Mathematical Sciences, Banaras Hindu University, Varanasi, 221005 India; 3 Department of Statistics, Banaras Hindu University, Varanasi, 221005 India; 4 Department of Surgical Oncology, Heritage Hospitals, Varanasi, 221005 India

**Keywords:** Lymphadenectomy, Ovarian neoplasm, Carcinoma ovarian epithelial, Meta-analysis

## Abstract

**Background:**

The debate surrounding systematic lymphadenectomy in the epithelial cancers of the ovary (EOC) was temporarily put to rest by the LION trial. However, there was a glaring disparity between the number of patients registered and the number of patients randomized suggesting inadvertent selection. A subsequent meta-analysis after this trial included all types of studies in the literature (randomized, non-randomized, case series, and, retrospective cohort), thus diluting the results.

**Methods:**

We conducted a meta-analysis of hazard ratios of randomized controlled trials, to study the role of systematic para-aortic and pelvic lymph node dissection in the EOC. A detailed search of MEDLINE, Cochrane, and Embase databases was done to look for the published randomized controlled trials (RCT) comparing lymphadenectomy versus no lymphadenectomy in EOC. A meta-analysis of hazard ratios (HR) was performed for overall survival (OS) and progression-free survival (PFS) using fixed and random effect models. The quality of the RCTs was evaluated on Jadad’s score, and the risk of bias was estimated by the Cochrane tool.

**Results:**

A total of 1342 patients with EOC were included for quantitative analysis. On meta-analysis, HR for PFS was 0.9 (95% CI 0.79–1.04) favoring lymphadenectomy. HR for OS was 1 (95% CI 0.84–1.18) signifying no benefit of systematic lymphadenectomy.

**Conclusion:**

The results show a trend towards increased PFS which did not reach statistical significance nor translate into any meaningful benefit in OS. There is still a need for a greater number of well-conducted, suitably powered trials to convincingly answer this question.

**Supplementary Information:**

The online version contains supplementary material available at 10.1186/s12957-022-02835-4.

## Background

Lymphadenectomy forms one of the tenets of cancer surgery. As defined by Siewert [1], radical surgery for cancer is defined as the resection of a tumor including the bed of the tumor in all three dimensions, along with lymph node dissection. Adequacy of lymph node dissection is defined by the achievement of the most favorable lymph node ratio (involved/dissected) of 0.2 or less. This finding is supported by SEER data analysis of 13,918 patients with ovarian epithelial carcinoma by Chan et al. [2], where they showed that the extent of lymphadenectomy and the number of positive nodes were significant independent predictors of survival.

The concept of lymph node dissection varies from organ to organ. Apart from achieving a complete resection and facilitating optimal debulking, lymph node dissection helps to stage the disease, which in turn may show increased survival due to stage migration [3].

Ovarian lymphatics primarily drain into the para-aortic and para-caval nodes and the lymphatics from the fallopian tubes and uterus drain to the pelvic nodes [4]. Hence, the lymph node dissection of these basins is fathomable in accordance with the general principles of cancer surgery. However, this relationship is not as simple for epithelial ovarian cancers.

A major debate in the surgical management of ovarian cancer remains the optimal management of the retroperitoneal and pelvic lymph nodes. The nodes appear normal on inspection but harbor the disease. Lymph nodes have been addressed using various approaches, ranging from no lymph dissection or sampling to berry picking of only enlarged nodes to systematic dissection of bilateral pelvic and paraaortic lymph nodes. Lymph node metastasis in epithelial ovarian malignancy has been reported in 44–53% of patients [5].

Several observational studies show that lymph node dissection has a distinct advantage in progression-free and overall survival [2, 6], in contrast to the findings of randomized trials, which show no benefit [5, 7].

Despite a very well-conducted randomized trial by Harter et al., wherein 647 node-negative patients with all stages of ovarian cancer were randomized to lymphadenectomy versus no lymphadenectomy, the results did not show improved overall or progression-free survival. However, the lymphadenectomy arm was found to have a higher incidence of postoperative complications. Subsequently, a meta-analysis by Chiyoda et al. [8] failed to answer the question. The trial had a highly selected patient population as reflected from the number of patients registered to the number of patients randomized and the meta-analysis considered all studies, including the retrospective and non-randomized ones, thus diluting the evidence. As a result, the NCCN recommends comprehensive staging in newly diagnosed early ovarian cancer, which includes para-aortic lymphadenectomy at least up to the inferior mesenteric artery and preferably up to the renal arteries [NCCN 2022 ver 1] [9]. However, for tumors greater than FIGO IIb, it recommends the removal of only the clinically positive nodes on imaging or those found intraoperatively, while resection of negative nodes is not recommended. The NCCN advice for early-stage cancer is based on textbook references, while the latter is based solely on the outcomes of a single RCT study [5]. The justification for advocating para-aortic lymphadenectomy in newly diagnosed early stage I–IIA disease seems to be upstaging to stage III in the case of a positive node, but not doing the procedure in clinically node-negative disease appears to have no influence on survival.

With the above background, we have conducted a meta-analysis of hazard ratios of randomized control trials to study the role of systematic para-aortic and pelvic lymph node dissection in epithelial cancers of the ovary.

## Methods

A detailed search of the literature was carried out in MEDLINE (PubMed), Embase, and Cochrane databases. We conducted a PubMed search using the following search string: (("ovarian neoplasms"[MeSH Terms] or ("ovarian"[All Fields] and "neoplasms"[All Fields]) or "ovarian neoplasms"[All Fields] or ("ovarian"[All Fields] and "cancer"[All Fields]) or "ovarian cancer"[All Fields]) and ("lymphnodal"[All Fields] or "lymphnode"[All Fields] or "lymphnodes"[All Fields] or ("lymph nodes"[MeSH Terms] or ("lymph"[All Fields] and "nodes"[All Fields]) or "lymph nodes"[All Fields] or ("lymph"[All Fields] and "node"[All Fields]) or "lymph node"[All Fields])) and ("dissect"[All Fields] or "dissected"[All Fields] or "dissecting"[All Fields] or "dissection"[MeSH Terms] or "dissection"[All Fields] or "dissections"[All Fields] or "dissects"[All Fields] or ("lymph node excision"[MeSH Terms] or ("lymph"[All Fields] and "node"[All Fields] and "excision"[All Fields]) or "lymph node excision"[All Fields] or "lymphadenectomies"[All Fields] or "lymphadenectomy"[All Fields]))) and (clinicaltrial[Filter]). The last search was done on January 22, 2022.

All the published randomized controlled trials (RCT) comparing lymphadenectomy versus no lymphadenectomy in epithelial ovarian cancers were included.

**Fig. 1:**
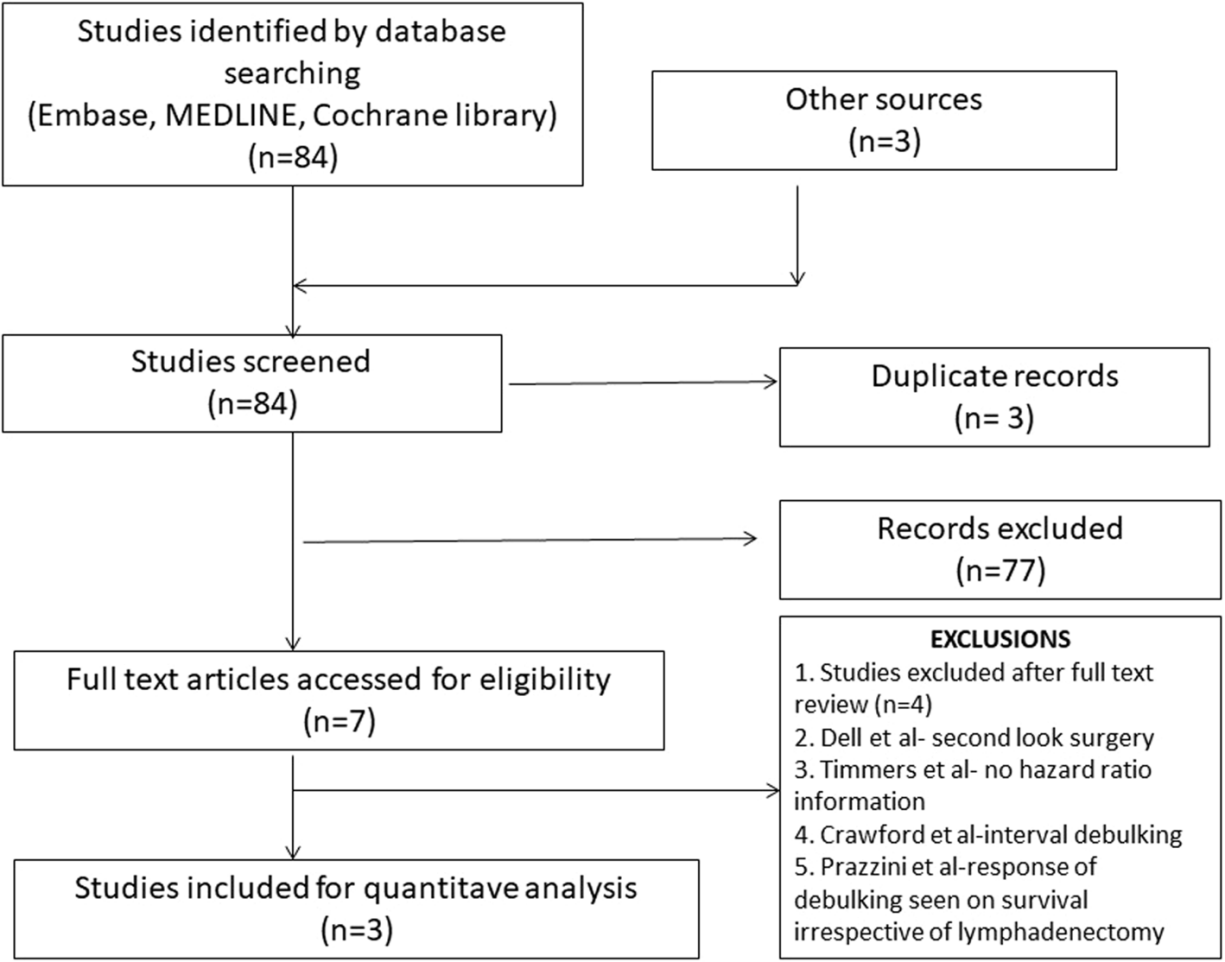
PRISMA diagram

Out of 84 studies found from the above search, 03 were shortlisted after abstract reviews [5, 7, 10] (Fig. 1). Additional crosschecks for any missing studies were done using manual search and back referencing. However, no more studies were found. In all, after the elimination of duplicates and exclusions, only three studies were considered for the final meta-analysis.

### Inclusion and exclusion criteria

All randomized trials that compared lymphadenectomy versus no lymphadenectomy in epithelial ovarian cancers were considered. Operability was considered based on preoperative evaluation of the respective centers and by the authors of each individual RCT.

Exclusion criteria entailed non-randomized studies, cohort, retrospective, observational studies, and unpublished abstracts presented in meetings.

### Data extraction

Two authors individually scanned all abstracts and shortlisted studies meeting the above inclusion criteria. Data from each of these shortlisted studies was collected on a pre-set proforma. Any discrepancies in the inclusion were settled after a discussion with a third member. The quality of each study was evaluated using Jadad’s score [11] and the revised Cochrane risk of bias tool (ROB 2.0 _IRPG_ beta v8) [12]. Patients with systematic lymphadenectomy and those without lymphadenectomy were categorized into two groups.

As different variables were evaluated in different studies, only the common variables were considered for the final analysis.

For final analyses, two endpoint variables were compared. The variables studied were progression-free survival (PFS) and overall survival (OS). The OS is defined as the time from the date of recruitment to death, and the PFS was defined as the time from recruitment to the progression of the disease, as described in the studies. Publication bias was measured using a funnel plot. One reference cited in the introduction and discussion is in German [1]. It was translated into English using Google Translate.

### Statistical analysis

As the data on individual patients were not available, we performed the meta-analysis of hazard ratio based on the methods described by Tierney et al. (2007) [13]. The statistical software R, especially the package “meta,” was used to perform the entire meta-analysis including the draws based on forest and funnel plots [14].

For OS and PFS data, we extracted the hazard ratio and its standard error based on different trials.

The Cox-Mantel estimate of the hazard ratio is formed by dividing the hazard rate under the treatment group by that under the control group. Obviously, the estimate so obtained measures the change in risk of treatment versus control over the follow-up period. Since the distribution of the log hazard ratio is nearly normal, one can consider logarithmic transformation for the purpose of meta-analysis. The formula for the hazard ratio can be given by

$$\textrm{H}{\textrm{R}}_{\textrm{CM}}=\frac{{\textrm{H}}_{\textrm{T}}}{{\textrm{H}}_{\textrm{C}}}=\frac{{}^{{\textrm{O}}_{\textrm{T}}}\!\left/ \!{}_{{\textrm{E}}_{\textrm{T}}}\right.}{{}^{{\textrm{O}}_{\textrm{C}}}\!\left/ \!{}_{{\textrm{E}}_{\textrm{C}}}\right.},$$

where *H*_*T*_ denotes the hazard rate under the treatment group and *H*_*C*_ denotes the hazard rate under the control group. Similarly, *O*_*T*_(E_T_) and *O*_*C*_(*E*_*C*_) denote the observed (expected) number of events (that is, deaths in our case) for treatment and control groups, respectively. One can refer to Parmar et al. [15] and Parmar and Machin [16] for further details.

As explained earlier, since the log hazard ratio is nearly normal, a confidence interval for the hazard ratio can be constructed by transforming it to the logarithmic scale and using the corresponding normal approximating formula given as follows:

$$\ln \left({\textrm{HR}}_{\textrm{CM}}\right)\pm {\textrm{z}}_{1-\frac{\upalpha}{2}}\left({\textrm{SE}}_{\ln \left({\textrm{HR}}_{\textrm{CM}}\right)}\right)$$

where ${\textrm{SE}}_{\ln \left({\textrm{HR}}_{\textrm{CM}}\right)}$ denotes the standard error of ln(HR_CM_). This can be further expressed as follows:

$${\textrm{SE}}_{\ln \left({\textrm{HR}}_{\textrm{CM}}\right)}=\sqrt{\frac{1}{{\textrm{E}}_{\textrm{T}}}+\frac{1}{{\textrm{E}}_{\textrm{C}}}}.$$

Once the interval is obtained, it can be transformed back to get the confidence interval for the hazard ratio.

An alternative estimate of the hazard ratio based on the Mantel-Haenszel estimator and the corresponding log-transformed confidence interval can be expressed as follows:

$${\textrm{HR}}_{\textrm{MH}}=\exp \left(\frac{{\textrm{O}}_{\textrm{T}}-{\textrm{E}}_{\textrm{T}}}{\textrm{V}}\right),$$

and

$$\ln \left({\textrm{HR}}_{\textrm{MH}}\right)\pm {\textrm{z}}_{1-\frac{\upalpha}{2}}\left({\textrm{SE}}_{\ln \left({\textrm{HR}}_{\textrm{MH}}\right)}\right),$$

where ${\textrm{SE}}_{\ln \left({\textrm{HR}}_{\textrm{MH}}\right)}$ denotes the standard error of HR_MH_,given by $\sqrt{\frac{1}{\textrm{V}}}$ and V is the Mantel-Haenszel hypergeometric variance.

In the absence of data for the individual patients, Tierney et al. [13] suggested methods available to obtain HRs and the associated summary statistics by carefully employing other existing data where the hazard ratio and its associated confidence interval (CI) were presented in a trial report. The variance of ln(HR), say V^∗^, can be obtained by the following:

$${V}^{\ast }=\frac{\ln \left(\textrm{upper}\ \textrm{limit}\ \textrm{of}\ \textrm{CI}\right)-\ln \left(\textrm{lower}\ \textrm{limit}\ \textrm{of}\ \textrm{CI}\right)}{2\times \left(z\ \textrm{score}\ \textrm{of}\ \textrm{upper}\ \textrm{CI}\ \textrm{boundery}\right)},$$

Obviously, if 95% CI is given in the trial report, the above equation leads to *V*^∗^ obtained as follows:

$${V}^{\ast }=\frac{\ln \left(\textrm{upper}\ \textrm{limit}\ \textrm{of}\ 95\%\textrm{CI}\right)-\ln \left(\textrm{lower}\ \textrm{limit}\ \textrm{of}\ 95\%\textrm{CI}\right)}{2\times 1.96}.$$

Likewise, *V* can also be obtained easily from the CI, if the same is desired. The test of significance, if required, can be performed using *z* test.

Sometimes, it is desired to perform a test for heterogeneity to examine the null hypothesis that all the studies are leading to the same effect. The heterogeneity is tested using chi-square and *I*^2^ statistics where the latter actually describes the percentage of variation across studies that is due to heterogeneity.

The manuscript is presented following the Preferred Reporting Items for Systematic Reviews and Meta-analysis (PRISMA) guidelines, and the PRISMA checklist has been provided in the supplemental material [17]. The meta-analysis has been registered with PROSPERO [18] (CRD42021281583).

## Results

**Fig. 2:**
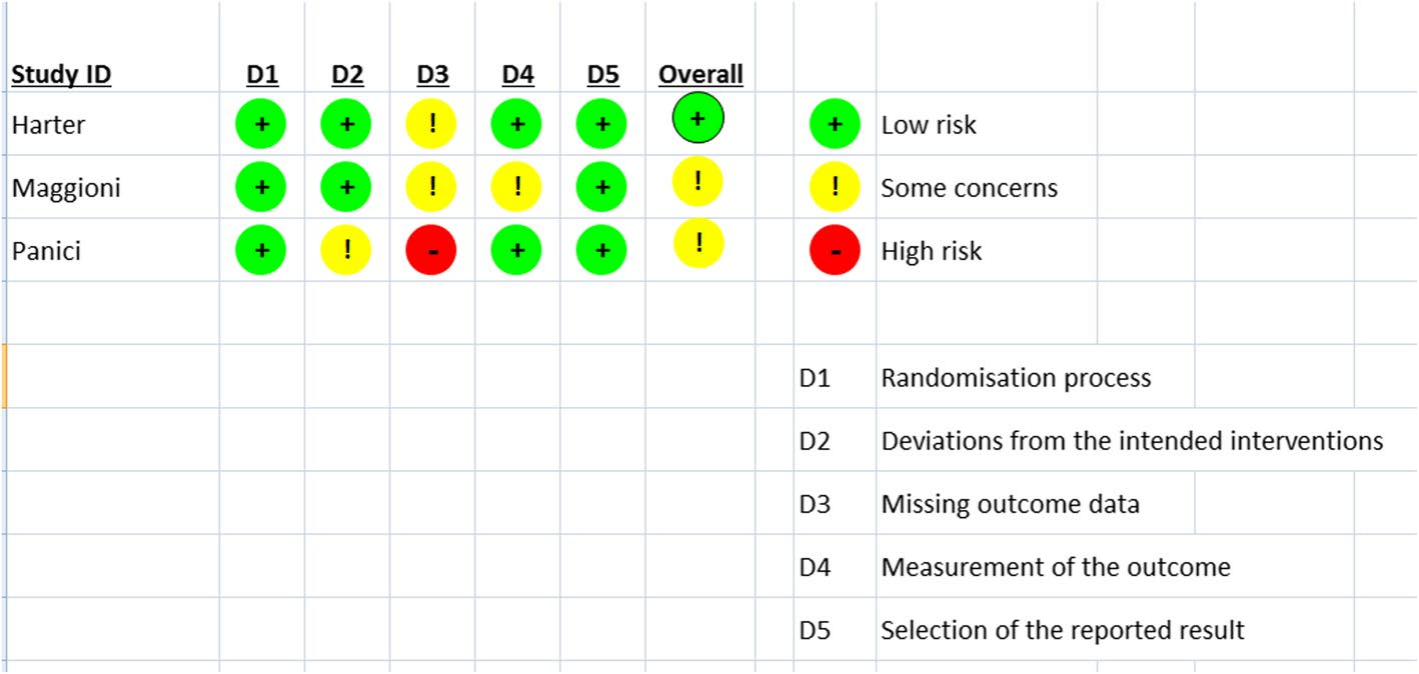
Cochrane risk of bias tool

Three randomized trials including 1342 patients were included for quantitative analysis. Of these 1342 patients, 677 underwent lymphadenectomy and 665 had no lymphadenectomy. The baseline characteristics of patients enrolled in different trials are shown in Table 1. The quality of evidence and risk bias were analyzed using Jadad’s score and the Cochrane risk bias tool, respectively (Table 1 and Fig. 2).

**Table 1:** Baseline characteristics

Parameters	Harter (2019)		Panici 2005		Maggioni 2006	
	Case *N*=323 *n* (%)	Control *N*=324 *n* (%)	Case *N*=216 *n* (%)	Control *N*=211 *n* (%)	Case *N*=138 *n* (%)	Control *N*=130 *n* (%)
Age (years) SD	60 21–83	60 23–78	53 45–61	56 47–62	51 43–60	52 44–59
Median CA 125	416	347	-	-	-	-
FIGO stage						
1–2A	15	17	-	-	135	129
2B–3A	41	52	-	-	-	-
3B–4	261	244	216	211	-	-
Missing data	6	11	-	-	3	1
Residual tumor						
None	321 (99.4)	322 (99.4)	80 (37)	79 (37.40)	133 ( 96.4)	126 (96.9)
<1cm	N M	NM	130 (60.2)	118 (55.9)	5 (3.6)	4 (3.1)
>1 cm	NM	NM	4 (1.9)	12 (5.7)	-	-
Missing data	NM	NM	2 (0.9)	2 (0.9)	-	-
Median number of resected nodes (number)						
Pelvic	22	-	28.5	1	24	3.5
Paraaortic	35	-	23	1	21	1
Both	57	-	51.5	4	47	5.5
Tumor grade						
1	-	-	19 (8.8)	11 (5.2)	30 (21.7)	20 (15.4)
2	-	-	50 ( 23.1)	37 (17.5)	29 (21)	41 (31.5)
3	-	-	142 (65.7)	160 (75.8)	72 (52.2)	65 (50)
Missing data	-	-	5 (2.3)	3 (1.4)	7 (5.1)	4 (3.1)
Cell type						
Serous	246 (73.1)	248 (76.6)	132 (62.6)	155 (71.8)	61 (44.2)	43 (33.1)
Endometriod	16 (4.9)	18 (4.5)	28 (13.3)	21 (9.6)	24 (17.4)	34 (26.2)
Mucinous	3 (0.9)	6 (1.9)	6 (2.8)	4 (1.9)	14 (10.1)	22 (16.9)
Clear cell	7 (2.2)	7 (2.2)	12 (5.7)	4 (1.9)	16 (11.6)	19 (14.6)
Undifferentiated	34 (10.5)	28 (8.6)	23 (10.9)	18 (8.3)	7 (5.1)	8 (6.1)
Other	15 (4.6)	14 (4.3)	8 (3.8)	12 (5.6)	8 (5.8)	2 (1.5)
Borderline tumor	2 (0.6)	3 (0.9)	-	-	-	-
Missing data	-	-	2 (0.9)	2 (0.9)	8 (5.8)	2 (1.5)
Jadad score (R+B+W)	(1+1) + (0) + (1)		(1+1) + (0) + (1)		(1+1) + (0) + (1)	

*NM* not mentioned, *R* randomisation, *B* blinding, *W* withdrawals

In all studies, lymphadenectomy showed an increased median time of surgery, increased median blood loss, increased chances of blood transfusion, and hospital stay duration, which were all statistically significant (Table 2). We did not analyze these parameters as all the trials unequivocally proved that the lymphadenectomy group had significantly increased perioperative complications and postoperative morbidity.

**Table 2:** Operative details

Parameters	Harter ( 2019)			Panici 2005			Maggioni 2006		
	Case *N*=323 *n* (%)	Control *N*=324 *n* (%)	*P* value	Case *N*=216 *n* (%)	Control *N*=211 *n* (%)	*P* value	Case *N*=138 *n* (%)	Control *N*=130 *n* (%)	*P* value
Median operating time (min)	340	280	<0.001	300	210	<0.001	240	150	< 0.001
Median blood loss (ml)	650	500	<0.001	1000	650	<0.001	600	300	< 0.001
Patients transfused (%)	63.7	56	0.005	71.7	59.2	0.006	35.5	21.85	0.012
Median hospital stay (days)	-	-	-	9	9	0.21	7	6	0.003
Postoperative admission to immediate or intensive care unit( %)	77.6	69	0.001	-	-	-	-	-	-
Infections treated with antibiotics (%)	25.8	18.6	0.03	-	-	-	-	-	-
Lymph cysts	24 (7.4)	1 (0.3)	< 0.001	14 (0.06)	0	-	8 (0.020	0	-
Repeat laparotomies for complications (%)	12.4	6.5	0.001	NM	NM	-	NM	NM	-
Death within 60 days	10 (3.1)	3 (0.9)	0.004	0	0	-	0	0	-
Postoperative systemic treatment	277 (85.7)	279 (86.1)	0.2	207 (960	198 (94)	0.3	77 (56)	78 (66)	0.11

*NM* not mentioned

### Progression-free survival

**Fig. 3:**
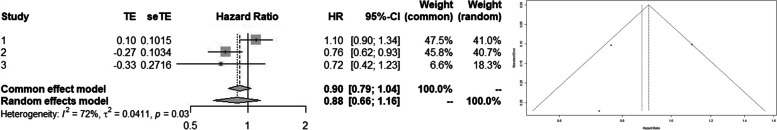
Forest and funnel plots comparing PFS in lymphadenectomy and no lymphadenectomy arms

On meta-analysis, the hazard ratio (HR) for PFS was 0.9 [95% CI 0.79–1.04] with the diamond almost shifted to the side favoring lymphadenectomy but failing to reach statistical significance (Fig. 3).

### Overall survival

**Fig. 4:**
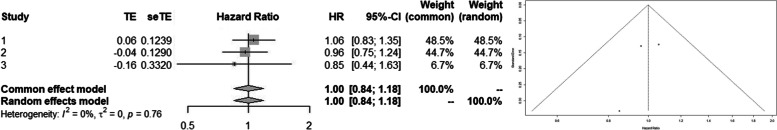
Forest and funnel plots comparing OS in lymphadenectomy and no lymphadenectomy arms

HR for overall survival was 1 [95% CI 0.84–1.18] with the diamond right in the center, signifying no benefit of systematic lymphadenectomy (Fig. 4).

## Discussion

The hazard ratio for overall survival in this meta-analysis was 1 [95% CI 0.84–1.18], which did not show any significance. These findings are similar to the most recently conducted meta-analysis by Chiyoda et al. [8], reporting a HR of 0.85 [95%CI 0.49–1.47]. The authors tried to justify this observation based on the narrative of the LION trial by Harter et al. [5], which stated that the effects of occult lymph node metastasis can be reversed by adjuvant chemotherapy. This hypothesis can be questioned by the results of studies by Burghardt et al. [19] and Baiocchi et al. [20] reporting 33.3–65.3% of patients having a residual disease in retroperitoneal nodes on post-chemotherapy second-look surgery and points towards the relative resistance of micro-metastatic disease present in retroperitoneal lymph nodes.

It will be very fascinating to know the effect of para-aortic node dissection in the present era of patients undergoing hyperthermic intraperitoneal chemotherapy (HIPEC) where the preferred pre-requisite is complete cytoreduction [21]. As noted by Siewert [1] in his favorable lymph node ratio and Carnino et al. [22], who stressed the importance of detecting more lymph node metastases when more lymph nodes are dissected, a meta-analysis by Bristow et al. [23] showed that a 10% increase in maximal cytoreduction was associated with a 5.5% increase in median survival. This study indicates that a complete cytoreduction independently affects survival. Hence, logically, systematic lymphadenectomy should lead to a more thorough cytoreduction, thus improving survival and providing better results with HIPEC. However, this is not reflected in the results.

The HR for PFS is 0.9 [95% CI 0.79–1.04], which shows a trend in favor of lymphadenectomy. These findings appear to differ from that of Chiyoda et al. [8], who showed an HR of 0.92 [95% CI 0.63–1.35]. This is mainly because Chiyoda et al. included only two trials [5, 10] of advanced ovarian cancer, whereas the present meta-analysis includes the third trial with the disease restricted to the pelvis [7]. We included this study because the lymphadenectomy arm in this study showed 79% involvement of para-aortic lymph nodes, making it stage III. We attribute this insignificant trend towards improved PFS to underpowered trials conducted so far. As mentioned in the introduction, even the trial conducted by Harter et al. [5] had a selected patient population as seen from the number of registered patients compared to the number of randomized patients.

One of the main concerns with any trials on lymphadenectomy is quality control. For over-enthusiastic surgeons, there is a possibility of dissecting in an area outside the required template. It may be seen while doing a pelvic node dissection where a surgeon can go well below the deep circumflex iliac vein into the inguinal region, leading to a false impression of adequate lymph node dissection on pathology without any consequent therapeutic benefit and instead leading to an increased incidence of postoperative morbidity. This is called contamination [24]. On the contrary, when lymph node stations are not dissected, which should have been dissected, leads to non-compliance [24]. Both these concepts are very well known and are dependent on the surgeon’s temperament. None of the trials included in the meta-analysis elaborate on any of these protocol violations.

Another confounder in stage III cancer of the ovary is whether the disease is stage III by virtue of the involvement of the non-pelvic peritoneum or due to retroperitoneal node involvement. As demonstrated by Carino et al. [22], there was significantly better survival in patients with stage III disease with only retroperitoneal lymph node involvement as compared to peritoneal disease (46 vs 12%, *p*=0.04). The point we are trying to make is that a patient with stage III peritoneal disease without any lymph node involvement will still have a worse prognosis even when an optimal cytoreduction with systematic lymphadenectomy has been done. Hence, we need to exclude or stratify such patients separately while designing any trial of lymphadenectomy for ovarian cancer.

Another dissent we have from the existing trials is that they did not consider, exclude, or stratify their patients based on histology or grade. Clear cell histology is well known in ovarian carcinoma, and its incidence ranges from 3 to 25% in various series [5, 7, 10, 25, 26]. There are no prospective studies on clear cell carcinoma, but when the results of various cohort and retrospective studies are analyzed [26, 27], it is seen that this is a relatively chemo-resistant and aggressive form of ovarian neoplasm. Its resistance to chemotherapy and aggressive biology further accentuates the role of systemic lymphadenectomy. Similarly, Carnino et al. [22] showed that grade 3 disease has a 49.1% chance of lymph node metastasis.

The importance of systematic lymph node dissection is also very much present in stage I ovarian cancer. It either upstages the disease in clinically stage I ovarian cancer, thus directing further treatment with adjuvant chemotherapy or, by adequate staging, obviates the need for unnecessary chemotherapy.

The uniqueness of this meta-analysis lies in the fact that this is the only meta-analysis of only randomized trials, thus making it evidence of the highest level. This is a meta-analysis of hazard ratios that utilizes a different methodology [13]. We have done a meta-analysis using the hazard ratios and not the number of events as done in most meta-analyses, hence making it statistically distinct. Lastly, we have analyzed ovarian carcinoma as one entity without classifying it as early or late. This is because the pelvic and abdominal spread is part of the same disease spectrum, and we can never know the actual stage of the disease till we examine the pelvic and para-aortic nodes.

The meta-analysis also has its limitations. The bias of individual studies forms the limitation of the meta-analysis itself. For example, randomization carried out by the two trials was before evaluating the feasibility of optimal debulking and the data about incompletely cytoreduced patients enrolled in the study is not known [7, 10].

We also do not know the impact systemic lymphadenectomy will have on patients undergoing hyperthermic intraperitoneal chemotherapy. Will that extra bit of disease clearance improve the outcomes of HIPEC?

## Conclusion

The authors acknowledge the trend of increased PFS, which has not reached significance nor translated into any meaningful benefit in OS. We still need a greater number of well-conducted, suitably powered trials to convincingly answer this question.

## Supplementary Information

**Additional file 1.** PRISMA checklist.

## Data Availability

All data generated and analyzed has been provided in the manuscript or in the supplementary file section.
